# Associations Between Behavior Change Techniques and Engagement With Mobile Health Apps: Protocol for a Systematic Review

**DOI:** 10.2196/35172

**Published:** 2022-03-29

**Authors:** Madison Milne-Ives, Sophie Homer, Jackie Andrade, Edward Meinert

**Affiliations:** 1 Centre for Health Technology University of Plymouth Plymouth United Kingdom; 2 School of Psychology Faculty of Health University of Plymouth Plymouth United Kingdom; 3 Department of Primary Care and Public Health School of Public Health Imperial College London London United Kingdom; 4 Harvard T H Chan School of Public Health Harvard University Boston, MA United States

**Keywords:** engagement, behavior change techniques, telemedicine, mobile apps

## Abstract

**Background:**

Digitally enabled care along with an emphasis on self-management of health is steadily growing. Mobile health apps provide a promising means of supporting health behavior change; however, engagement with them is often poor and evidence of their impact on health outcomes is lacking. As engagement is a key prerequisite to health behavior change, it is essential to understand how engagement with mobile health apps and their target health behaviors can be better supported. Although the importance of engagement is emphasized strongly in the literature, the understanding of how different components of engagement are associated with specific techniques that aim to change behaviors is lacking.

**Objective:**

The purpose of this systematic review protocol is to provide a synthesis of the associations between various behavior change techniques (BCTs) and the different components and measures of engagement with mobile health apps.

**Methods:**

The review protocol was structured using the PRISMA-P (Preferred Reporting Items for Systematic Reviews and Meta-Analyses Protocols) and the PICOS (Population, Intervention, Comparator, Outcome, and Study type) frameworks. The following seven databases will be systematically searched: PubMed, Embase, Cumulative Index to Nursing and Allied Health Literature, APA PsycInfo, ScienceDirect, Cochrane Library, and Web of Science. Title and abstract screening, full-text review, and data extraction will be conducted by 2 independent reviewers. Data will be extracted into a predetermined form, any disagreements in screening or data extraction will be discussed, and a third reviewer will be consulted if consensus cannot be reached. Risk of bias will be assessed using the Cochrane Collaboration Risk of Bias 2 and the Risk Of Bias In Non-Randomized Studies - of Interventions (ROBINS-I) tools; descriptive and thematic analyses will be conducted to summarize the relationships between BCTs and the different components of engagement.

**Results:**

The systematic review has not yet started. It is expected to be completed and submitted for publication by May 2022.

**Conclusions:**

This systematic review will summarize the associations between different BCTs and various components and measures of engagement with mobile health apps. This will help identify areas where further research is needed to examine BCTs that could potentially support effective engagement and help inform the design and evaluation of future mobile health apps.

**Trial Registration:**

PROSPERO CRD42022312596; https://tinyurl.com/nhzp8223

**International Registered Report Identifier (IRRID):**

PRR1-10.2196/35172

## Introduction

### Background

This systematic review aims to provide an overview of how behavior change techniques (BCTs) [1] are associated with different components of engagement with mobile health apps. Effective engagement with digital health interventions is an essential factor influencing their ability to support positive behavior change. Although several models and frameworks conceptualizing engagement and its association with intervention impact have recently been published, a comprehensive understanding of how to develop digital health interventions that significantly impact health behavior and outcomes is still lacking [2]. This is a serious concern because although mobile health apps are frequently used to deliver health behavior change interventions [3], there is still a lack of evidence supporting their impact on behavior and health outcomes [4,5]. This lack of evidence necessitates an in-depth examination of the stages of engagement and behavior change so that particular barriers and blockers can be targeted. BCTs, “observable, replicable, and irreducible components” of behavior change interventions [1], provide a means of reliably classifying and testing potential strategies for altering behavior to address particular barriers. Understanding the associations between different BCTs, theoretical components of engagement, and measures of engagement will provide insight into how BCTs can be incorporated to improve and personalize the design of digital health interventions to support effective engagement.

Engagement with digital health interventions can be poor, which limits their potential impact. As health care service delivery is becoming increasingly digital and accessible through personal devices like smartphones and wearables [6,7], there is a need to ensure that these digital interventions are achieving their intended outcomes. The potential impact of digital interventions is limited by the extent of users’ engagement with them [8-10]; a meta-analysis of engagement with digital mental health interventions found a significant positive association between engagement and mental health outcomes [11]. However, the variety in the definitions and measures of engagement means that reliable quantitative estimates of the relationship between engagement and outcomes are still lacking [8,11]. Maintaining engagement with digital health interventions is a common challenge. Studies on engagement with mobile health apps and wearable devices often observe poor long-term use [9,12,13] and high rates of attrition [8,14]. Although the duration of use is a commonly used indicator of engagement with a digital health intervention, its validity has been questioned because it only captures 1 component of engagement [2,10].

**Figure 1:**
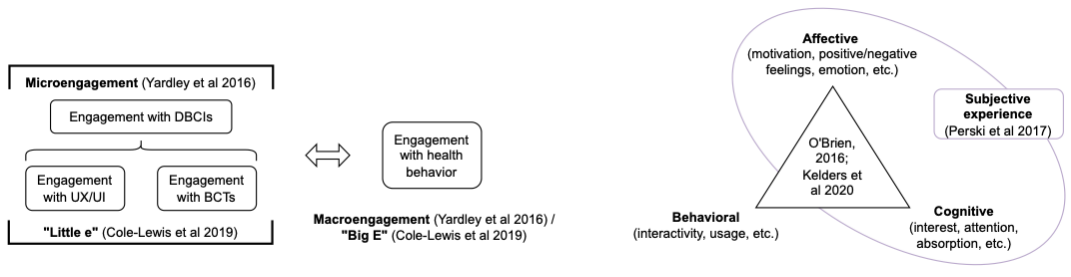
Summary of key theoretical concepts of engagement with digital health [15-17,20,21]. BCT: behavior change technique; DBCI: digital behavior change intervention; UI: user interface; UX: user experience.

Inconsistency in the way engagement is defined and measured is one of the challenges associated with studying engagement [15,16]. The lack of a clear, comprehensive, and well-accepted conceptualization of engagement is a major gap, which several papers and reviews have recently tried to address [2,15,17,18]. Although various models and definitions of engagement have been proposed, there is a general consensus that engagement is a multifaceted concept [15-17,19,20]. These conceptual frameworks highlight the importance of considering cognitive, behavioral, and affective aspects of engagement [10,15,17,20], as well as examining different levels of engagement with digital behavior change interventions (DBCIs) and health behaviors [16,19] (see Figure 1). A key review defined engagement in terms of 2 key components, extent of usage and subjective experience [17]. Another paper emphasizes the importance of the relationship between engagement with the intervention and the target behavior by defining “effective engagement” as the level of engagement sufficient to achieve the aims of the intervention [21]. This highlights the crucial distinction between engagement with the intervention and engagement with the behavior, as frequent or indefinite engagement with the intervention may not be required to support sustained engagement with the behavior, as shown in Figure 1.

Engagement with the intervention can be subdivided into engagement with the device or software and engagement with BCTs or “active ingredients” of the intervention [16,22] (see Figure 1, far left). As the DBCI is the proposed trigger for the behavior change, engagement with the health behavior is thought to depend on engagement with the DBCI [16,21]. However, the interconnected nature of engagement with the device, BCTs, and behavior makes it challenging to untangle the relationships between various stages and components of engagement and different BCTs. This is because BCTs can be used to influence users’ engagement with the health behavior, for example, by including goal setting (BCT 1.1) or self-monitoring of behavior (BCT 2.3) features to support users’ engagement with physical activity. However, BCTs can also provide “feedback” to influence engagement with devices or with other BCTs included in the DBCI, for example, by using prompts or cues (BCT 7.1) such as app notifications to remind a user to engage with the app or with specific BCT-based features on the app.

Different BCTs are associated with different theoretical barriers to behavior (eg, capability, opportunity, and motivation) [23,24]. For instance, “instruction on how to perform the behavior” (BCT 4.1) is commonly used to support a “training” intervention function, which in turn can target barriers related to physical and psychological capability [24]. Given the different functions associated with BCTs, it seems likely that different BCTs will also have different relationships with the 3 main components of engagement (affective, cognitive, and behavioral). To improve engagement with DBCIs and target behaviors, it is essential to understand the relationships between BCTs and the various components of engagement and incorporate them into the design and evaluation of digital health interventions.

### Rationale

The growing recognition of the importance of engagement in the design and evaluation of digital health interventions has led to an exponential increase in research concerning that topic in recent years. Given the accepted importance of engagement as a prerequisite for behavior change [17], several systematic reviews have examined various factors that could influence engagement with digital health interventions [25-28]. Among these, the analysis in 1 review [25] is structured around the COM-B (Capability, Opportunity, Motivation – Behavior) model, which is part of the Behavior Change Wheel theoretical framework [23]. The authors identified 26 different factors relating to capability, opportunity, and motivation that have been associated with uptake of and engagement with mobile health apps in the literature [25]. This provides a valuable, theory-based contribution to the understanding of factors affecting engagement with mobile health apps. However, despite including studies with either qualitative or quantitative (primarily system use data) measures of engagement and using a multifaceted definition of engagement [17], the review did not clarify how the influence of these factors varied for the different components and measures of engagement.

The importance of understanding the factors associated with engagement lies in their potential to inform designs that improve “effective engagement” with DBCIs and thereby better support behavior change and the associated positive health outcomes. Because engagement is a complex and multifaceted concept, it is important to understand how specific BCTs are related to different elements of engagement and which ones have the most influence on effective engagement and health outcomes [21]. As the best strategies for achieving effective engagement could differ among individuals, an understanding of how different BCTs are associated with different components of engagement would enable digital health interventions to be personalized to individuals, specific populations, or contexts, providing an opportunity to increase their health impact.

PROSPERO was searched using various combinations of the following keywords: engagement, digital health interventions, DBCIs, behavior change techniques, BCT, mobile health apps, mHealth, eHealth, and digital behavior change. None of the registered protocols aimed to examine the associations between BCTs and the different components of engagement; however, the search terms identified the PROSPERO preregistration for one of the previous reviews cited in this rationale [25], indicating that the search terms were appropriate.

### Objectives

The main aim of the review is to provide a synthesis of the associations between BCTs and the different components of engagement (and their outcome measures) with mobile health apps in the literature. The following are the key objectives of this review: (1) to identify the BCTs being incorporated in the development of mobile health apps; (2) to identify the components of engagement that are being evaluated in studies on mobile health apps and how the different components are being measured; (3) to document the associations between specific BCTs and engagement component outcomes and outcome measures; and (4) to compare those associations across the included studies to hypothesize causal relationships between specific BCTs and specific components of engagement that can be empirically evaluated in future studies.

## Methods

### Overview

The PRISMA-P (Preferred Reporting Items for Systematic Reviews and Meta-Analyses Protocols) [29] and the PICOS (Population, Intervention, Comparator, Outcome, Study type) frameworks [30,31] will be used to structure this review and develop the search strategy. The PRISMA-P checklist is available in Multimedia Appendix 1. This review is registered on PROSPERO (registration number: CRD42022312596).

### Eligibility Criteria

The PICOS framework is based on the research questions and is presented in Table 1.

**Table 1:** PICOS (Population, Intervention, Comparator, Outcome, and Study type) framework.

Framework component	Description
Population	Mobile health app users of any age (adults and children)
Intervention	Mobile health apps that explicitly use BCTs^a^ in their design to target at least 1 of 5 key health categories established in the literature, including drug use, alcohol use, diet, physical activity, and mental health
Comparator	No comparator is required.
Outcomes	The primary outcome will be the qualitative or quantitative engagement outcomes measured (including any components of engagement specified by a theoretical framework). Secondary outcomes will include the BCTs included in the mobile health app, the measure(s) of engagement used by the study, and the behavioral and health outcomes reported.
Study types	Studies that evaluate engagement with at least 1 mobile health app that uses BCTs will be eligible (including randomized controlled trials, quantitative, qualitative, cohort, and case studies). Reviews, protocols, papers that describe interventions without evaluating them, and papers where full texts cannot be identified (eg, conference abstracts) will be excluded.

^a^BCT: behavior change technique.

### Search Strategy

The search will be conducted in seven databases: PubMed, Embase, Cumulative Index to Nursing and Allied Health Literature, APA PsycInfo, ScienceDirect, Cochrane Library, and Web of Science. These databases were chosen because they were commonly searched in previous systematic reviews relating to engagement and digital health interventions, and they broadly cover topics related to digital technology, health, and behavior change. Keywords and MeSH (Medical Subject Headings) terms relating to engagement with digital health behavior change interventions were identified in an initial review of the literature and used to develop the search strategy. These search terms were expanded upon and grouped into three themes (see Table 2) to develop the following search structure: engagement (MeSH OR Keywords) AND mobile health apps (MeSH OR Keywords) AND behavior change (MeSH OR Keywords). Sample searches conducted in PubMed, Embase (Ovid), and Web of Science are included in Multimedia Appendix 2.

**Table 2:** Search terms.

Category	MeSH^a^	Keywords (in title or abstract)
Engagement	Treatment Adherence and Compliance OR Patient Participation OR Patient Compliance	Engagement OR adherence OR compliance OR maintenance OR acceptability OR satisfaction OR attention OR enjoyment OR interest OR affect OR flow OR “cognitive absorption” OR “subjective experience” OR immersion OR presence OR ((amount OR frequency OR duration OR depth OR breadth) NEAR/2 (use OR usage)) OR dose OR stickiness OR dropout OR “drop out” OR “drop-out” OR attrition
Mobile health apps	Telemedicine OR Mobile Applications	“mHealth” OR “mobile health” OR “eHealth” OR telehealth OR ((mobile OR phone OR smartphone OR cell OR mHealth OR “behavior change” OR “behavior change” OR digital) NEAR/2 (app OR apps OR application*))
Behavior change	Behavior Control	“behavior change techniques” or “behavior change techniques” or “BCT” or “behavior change technique” or “behavior change technique” or “behavioral change strategies” or “behavioral change strategies” or “behavior change wheel” or “behavior change wheel” or “behavioral theory” or “behavioral theory” or “behavior change theory” or “behavior change theory” or “health behavior change” or “behavior change” or “behavior change” or “digital behavior change intervention” or “digital behavior change intervention” or “DBCI” or “behavior change intervention”

^a^MeSH: Medical Subject Headings.

### Inclusion Criteria

The review will include studies that evaluate theory-based mobile apps for health behavior change. Studies will be included if they evaluate at least 1 component or measure of engagement (quantitative or qualitative) with a mobile app that uses BCTs to influence health behavior. No restrictions will be placed on the type of health behavior or the sample population examined in the initial screening to ensure that all eligible studies are identified. If there are too many studies eligible after initial screening to conduct a thorough review, the number of studies will be restricted based on health behavior. This will limit included studies to those that focus on at least 1 of 5 key health categories, including drug use, alcohol use, diet, physical activity, and mental health [32,33], aligned with a previous review by the authors [5]. Studies with any type of sampled population will be eligible for inclusion, with no restrictions on age, gender, or country. Interventions with comparisons to control groups with no intervention, waiting list or irrelevant interventions, minimal interventions, usual care, other mobile apps, telemedicine, and internet-based or in-person interventions will be included. Studies with no comparators will also be included.

### Exclusion Criteria

Studies involving mobile health apps that do not detail the BCTs included in the app will be excluded from the review. Studies that do not evaluate at least 1 measure of engagement, such as reviews, protocols, papers that describe interventions without evaluating them, and papers where full texts cannot be identified (eg, conference abstracts) will also be excluded.

### Screening and Article Selection

The references returned by each database search will be exported into the citation management software EndNote X9 (Clarivate) so that duplicate references can be identified and removed. The screening will take place in three stages: (1) Keywords based on the search criteria will be entered into EndNote’s search function over multiple passes to exclude any studies that are clearly ineligible (eg, protocols, reviews). (2) The titles and abstracts of the remaining references will be screened by 2 independent reviewers. (3) The full texts of the studies will be screened by 2 independent reviewers to determine the final set of included papers. Any disagreements between reviewers will be discussed until consensus; if consensus cannot be reached, a third reviewer will be consulted. Details of the screening and selection process will be recorded in a PRISMA flow diagram to ensure study reproducibility and the EndNote searches in stage 1 will be recorded and included in the review as an appendix.

### Data Extraction

The full texts of all the articles included in the final set will be read by 2 independent reviewers to extract the required data mentioned in Table 3. As with the screening process, any disagreements will be discussed and resolved by involving a third reviewer if necessary.

**Table 3:** Article information and data extraction.

Article information	Data to be extracted
General study information	Year of publication Country of study Sample demographics (including age, gender, target population) Initial/intended sample size Analyzed sample size Study duration
Intervention	App name Operating platform (eg, iOS, Android) Target health behavior Specific aim of the intervention Behavioral theory used in the design of the app (if any) How the app was developed (eg, iterative design, experience-based co-design, etc) Number of included behavior change techniques [1] List of included behavior change techniques [1] Intended purpose of included behavior change techniques (if specified) Intended use (eg, dose and duration if specified)
Evaluation	Component(s) of engagement examined Engagement outcome measures Effect of intervention on engagement outcomes (including engagement with specific behavior change techniques, the app, and the target health behavior) Effect of intervention on behavior change outcomes Effect of intervention on participant health outcomes

### Quality Appraisal and Risk of Bias Assessment

The risk of bias of the studies will be evaluated by 2 independent reviewers using the Cochrane Collaboration Risk of Bias 2 tool for randomized controlled trials [34,35] and the Risk Of Bias In Non-Randomized Studies - of Interventions (ROBINS-I) tool for nonrandomized studies [36]. The GRADE (Grading of Recommendations, Assessment, Development and Evaluations) guidelines will be used to assess the strength of the body of evidence gathered during the review [37].

### Data Analysis and Synthesis

The feasibility of conducting a meta-analysis will be examined when the data are extracted; however, a meta-analysis may not be possible owing to the expected variety of study aims, measures, and reported outcomes. The extracted data will be summarized by conducting a descriptive analysis to provide counts of the engagement components examined, outcome measures used, health behaviors targeted, and levels of evidence showing the effectiveness of BCTs for engagement, behavioral, and health outcomes. The associations between the inclusion of various BCTs and evidence of their effectiveness for various outcomes will be mapped. Any qualitative data reported will be examined by performing a thematic analysis to provide contextual data about the potential relationships between BCTs and certain components of engagement. The risk of bias in the studies will be considered in the synthesis.

## Results

The full systematic review has not yet started, but it is expected to be completed and submitted for publication by May 2022.

## Discussion

A systematic review of the literature on engagement with theoretically based mobile apps for health behavior change will contribute to the understanding of how BCTs fit into the multifaceted state and process of engagement. With the ubiquity of mobile health apps and the continuous growth of digitally enabled care [6], it is necessary to ensure that the mobile health apps being used are effective. A key component of the efficacy of DBCIs is the extent to which the user engages effectively with the intervention to achieve the intended target behavior. An overview of the associations between BCTs and the different components and measures of engagement will inform the design and evaluation of mobile health apps. Based on the data, we will determine what conclusions can be drawn, identify the limitations of our systematic review, and propose key topics for future research.

## Appendix

Multimedia Appendix 1 PRISMA-P (PRISMA-P: Preferred Reporting Items for Systematic Reviews and Meta-Analyses Protocols) checklist.

Multimedia Appendix 2 Sample search strings.

## Abbreviations

BCT: behavior change technique
DBCI: digital behavior change intervention
MeSH: Medical Subject Headings
PICOS: Population, Intervention, Comparator, Outcome, and Study type
PRISMA-P: Preferred Reporting Items for Systematic Reviews and Meta-Analyses Protocols
ROBINS-I: Risk Of Bias In Non-Randomized Studies - of Interventions
