# Supplementary material for: Associations Between Behavior Change Techniques and Engagement With Mobile Health Apps: Protocol for a Systematic Review
Source: JMIR Res Protoc. 2022 Mar 29;11(3):e35172. doi: 10.2196/35172 (PMC9006128; doi:10.2196/35172)
Supplement: Multimedia Appendix 2 [file resprot_v11i3e35172_app2.docx]

### Appendix 2. Sample search strings

| Database | Search string | References |
| --- | --- | --- |
| PubMed^a^ | ((Treatment Adherence and Compliance OR Patient Participation OR Patient Compliance[MeSH Terms]) OR (Engagement[Title/Abstract] OR adherence[Title/Abstract] OR compliance[Title/Abstract] OR maintenance[Title/Abstract] OR acceptability[Title/Abstract] OR satisfaction[Title/Abstract] OR attention[Title/Abstract] OR enjoyment[Title/Abstract] OR interest[Title/Abstract] OR affect[Title/Abstract] OR flow[Title/Abstract] OR "cognitive absorption"[Title/Abstract] OR "subjective experience"[Title/Abstract] OR immersion[Title/Abstract] OR presence[Title/Abstract] OR (amount[Title/Abstract] AND (use[Title/Abstract] OR usage[Title/Abstract])) OR (frequency[Title/Abstract] AND (use[Title/Abstract] OR usage[Title/Abstract])) OR (duration[Title/Abstract] AND (use[Title/Abstract] OR usage[Title/Abstract])) OR (depth[Title/Abstract] AND (use[Title/Abstract] OR usage[Title/Abstract])) OR (breadth[Title/Abstract] AND (use[Title/Abstract] OR usage[Title/Abstract])) OR dose[Title/Abstract] OR stickiness[Title/Abstract] OR dropout[Title/Abstract] OR "drop out"[Title/Abstract] OR "drop-out"[Title/Abstract] OR attrition[Title/Abstract])) AND ((Telemedicine OR Mobile Applications[MeSH Terms]) OR ("mHealth"[Title/Abstract] OR "mobile health"[Title/Abstract] OR "eHealth"[Title/Abstract] OR telehealth[Title/Abstract] OR (mobile[Title/Abstract] AND (app[Title/Abstract] OR apps[Title/Abstract] OR application*[Title/Abstract])) OR (phone[Title/Abstract] AND (app[Title/Abstract] OR apps[Title/Abstract] OR application*[Title/Abstract])) OR (smartphone[Title/Abstract] AND (app[Title/Abstract] OR apps[Title/Abstract] OR application*[Title/Abstract])) OR (cell[Title/Abstract] AND (app[Title/Abstract] OR apps[Title/Abstract] OR application*[Title/Abstract])) OR (mHealth[Title/Abstract] AND (app[Title/Abstract] OR apps[Title/Abstract] OR application*[Title/Abstract])) OR ("behaviour change"[Title/Abstract] AND (app[Title/Abstract] OR apps[Title/Abstract] OR application*[Title/Abstract])) OR (Behavior Control[MeSH Terms]) OR "behavior change"[Title/Abstract] AND (app[Title/Abstract] OR apps[Title/Abstract] OR application*[Title/Abstract])) OR (digital[Title/Abstract] AND (app[Title/Abstract] OR apps[Title/Abstract] OR application*[Title/Abstract])))) AND ("behaviour change techniques"[Title/Abstract] OR "behavior change techniques"[Title/Abstract] OR "BCT"[Title/Abstract] OR "behaviour change technique"[Title/Abstract] OR "behavior change technique"[Title/Abstract] OR "behavioral change strategies"[Title/Abstract] OR "behavioural change strategies"[Title/Abstract] OR "behaviour change wheel"[Title/Abstract] OR "behavior change wheel"[Title/Abstract] OR "behavioural theory"[Title/Abstract] OR "behavioral theory"[Title/Abstract] OR "behaviour change theory"[Title/Abstract] OR "behavior change theory"[Title/Abstract] OR "health behaviour change"[Title/Abstract] OR "behaviour change"[Title/Abstract] OR "behavior change"[Title/Abstract] OR "digital behaviour change intervention"[Title/Abstract] OR "digital behavior change intervention"[Title/Abstract] OR "DBCI"[Title/Abstract] OR "behaviour change intervention"[Title/Abstract])) | 1399 |
| Web of Science^b,c^ | ("Treatment Adherence and Compliance" OR "Patient Participation" OR "Patient Compliance" ofengagement OR adherence OR compliance OR maintenance OR acceptability OR satisfaction OR attention OR enjoyment OR interest OR affect OR flow OR "cognitive absorption" OR "subjective experience" OR immersion OR presence OR ((amount OR frequency OR duration OR depth OR breadth) NEAR/2 (use OR usage)) OR dose OR stickiness OR dropout OR "drop out" OR "drop-out" OR attrition) AND (Telemedicine OR "Mobile Applications" OR "mHealth" OR "mobile health" OR "eHealth" OR telehealth OR ((mobile OR phone OR smartphone OR cell OR mHealth OR "behaviour change" OR "behavior change" OR digital) NEAR/2 (app OR apps OR application*))) AND ("behavior control" OR “behaviour change techniques” OR “behavior change techniques” OR “BCT” OR “behaviour change technique” OR “behavior change technique” OR "behavioral change strategies" OR "behavioural change strategies" OR "behaviour change wheel" OR "behavior change wheel" OR "behavioural theory" OR "behavioral theory" OR "behaviour change theory" or "behavior change theory" OR "health behaviour change" OR "behaviour change" OR "behavior change" OR "digital behaviour change intervention" OR "digital behavior change intervention" OR "dbic" OR "behaviour change intervention") | 1002 |
| Embase (Ovid) | ((patient compliance/ or patient participation/) or ((Engagement or adherence or compliance or maintenance or acceptability or satisfaction or attention or enjoyment or interest or affect or flow or "cognitive absorption" or "subjective experience" or immersion or presence or ((amount or frequency or duration or depth or breadth) adj2 ("use" or usage)) or dose or stickiness or dropout or "drop out" or "drop-out" or attrition).ti,ab.)) AND ((telemedicine/ or mobile application/) or ("mHealth" or "mobile health" or "eHealth" or telehealth or ((mobile or phone or smartphone or cell or mHealth or "behaviour change" or "behavior change" or digital) adj2 ("app" or apps or application*)).ti,ab.)) OR ((behavior control/) or ("behaviour change techniques" or "behavior change techniques" or "BCT" or "behaviour change technique" or "behavior change technique" or "behavioral change strategies" or "behavioural change strategies" or "behaviour change wheel" or "behavior change wheel" or "behavioural theory" or "behavioral theory" or "behaviour change theory" or "behavior change theory" or "health behaviour change" or "behaviour change" or "behavior change" or "digital behaviour change intervention" or "digital behavior change intervention" or "DBCI" or "behaviour change intervention").ti,ab.)) | 645 |

^a^PubMed does not support proximity operators, so the terms joined with proximity operators have instead been joined with ‘AND’

^b^Web of Science does not use MeSH terms, so all MeSH terms have been included as keywords.

^c^Keywords searched in ‘Topic,’ which includes title, abstract, author keywords, and Keywords Plus.
